# Status of and Factors Influencing the Stigma of Chinese Young and Middle-Aged Maintenance Hemodialysis Patients: A Preliminary Study

**DOI:** 10.3389/fpsyg.2022.873444

**Published:** 2022-05-11

**Authors:** Nina Zhang, Fengxia Lai, Yong Guo, Lan Wang

**Affiliations:** ^1^Department of Nursing, Shanghai Jiao Tong University Affiliated Sixth People’s Hospital, Shanghai, China; ^2^School of Nursing, Medical College of Soochow University, Suzhou, China; ^3^Department of Critical Care Medicine, Shanghai Jiao Tong University Affiliated Sixth People’s Hospital, Shanghai, China

**Keywords:** young and middle-aged, hemodialysis, stigma, influencing factors, status

## Abstract

Many young and middle-aged maintenance hemodialysis patients suffer a poor prognosis, experience a series of problems during long-term treatment and are thus prone to stigma. This study was designed to analyze stigma in young and middle-aged maintenance hemodialysis patients and explore its influencing factors. This study was conducted as a cross-sectional descriptive study with a convenience sampling method and included 97 patients from Shanghai Jiao Tong University Affiliated Sixth People’s Hospital between November 2020 and February 2021. The Social Impact Scale, a demographic questionnaire, and hemodialysis indicators were used in the investigation. Patient biochemical indexes from hemodialysis were compared. Young and middle-aged maintenance hemodialysis patients had a medium level of stigma. Patients who had low income, were younger, were male, had long-term hemodialysis and were unemployed had significantly higher stigma scores than other patients (*P* < 0.05). Age, gender, occupational status, annual household income and the duration of hemodialysis were found to be the main factors related to stigma in young and middle-aged maintenance hemodialysis patients by multiple regression analysis. Perceptions of hemodialysis-related stigma were common in our sample. Patients who had low income, were younger, were male, had long-term hemodialysis and were employed had a higher level of stigma, which deserves attention from clinical medical workers. Replication studies are needed to confirm these findings.

## Introduction

Stigma, which originates from the Greek word “stizein,” is a negative perception that something about a person is abnormal, out of the ordinary or bad (Goffman, 1963). Self-stigma arises in patients living in a discriminatory social environment, and because they agree with the fixed stereotypes of society, they then internalize these stereotypes into their own subjective prejudice and negative cognitive experience (Fox et al., 2018). Self-stigma may lead to treatment avoidance or diminished treatment adherence in patients with chronic illnesses (Oexle et al., 2018; Schnyder et al., 2017). Therefore, stigma has become a predictor of patients’ disease adaptability and quality of life and has attracted the attention of psychologists and medical workers worldwide.

With the worldwide incidence of renal failure caused by chronic kidney disease and other chronic diseases continuing to rise, the number of hemodialysis patients is increasing year by year. Recent epidemiological studies have found that the population of young and middle-aged maintenance hemodialysis (MHD) survivors has gradually increased (Zhang et al., 2012). MHD is one of the most important treatments for patients with end-stage renal disease. Dialysis treatment requires patients to return to the hospital two or three times a week for lengthy periods to complete treatment. The requirements of MHD treatment disrupt the normal rhythm of the lives of young and middle-aged MHD patients, making them prone to disconnect from society (Zhang et al., 2012).

In addition, most MHD patients have a series of symptoms and complex complications, such as fatigue, nausea, vomiting, pruritus, body aches, chest pain, cramps, hypotension, hypertension, acute hemolysis, dialysis imbalance syndrome, and painful muscle spasms (Yu et al., 2012; Masud et al., 2018; Sattar et al., 2016). These symptoms and complications not only cause physical discomfort but also reduce the physiological function of MHD patients, resulting in a decline in patients’ quality of life (Wang et al., 2017). Moreover, these symptoms and complications can be controlled only through drug treatment and dietary restrictions, which also increases the economic burden on MHD patients (Howell et al., 2019). In addition to physical symptoms, Carolina et al. also found that 60.3% of MHD patients have psychological symptoms such as depression and anxiety (Pretto et al., 2020). These negative emotions lead to patients’ unwillingness to participate in social activities, low self-esteem and guilt toward their families (Hagren et al., 2005). In short, the decline in physical function, the change in living environment and economic status and the emergence of psychological problems caused by long-term hemodialysis treatment make MHD patients prone to stigma (Lee et al., 2021).

Although some studies have mentioned that MHD patients have a sense of stigma, there are few studies on the level of stigma of MHD patients, especially young and middle-aged MHD patients. In addition, some studies have found that some hemodialysis indicators, such as dialysis adequacy, are positively correlated with the nutritional status and quality of life of MHD patients (Teixeira Nunes et al., 2008; Finkelstein and Foo, 2020). Dialysis indicators can reflect the adequacy of dialysis and the levels of important blood components, such as hemoglobin, calcium and phosphorus in patients. Adequate hemodialysis is beneficial to eliminate metabolic toxins in the human body and improve physical condition. Inadequate dialysis often causes symptoms such as anorexia and malnutrition, and malnutrition is an important factor in the decline of quality of life and the increase in mortality and hospitalization rate in MHD patients (Guo et al., 2017). The physiological state reflected by dialysis indicators affects the physical and mental state of patients as they face other people in life, which may further affect the stigma experienced by patients. However, there are few studies on the impact of dialysis indicators on the stigma of MHD patients. This study aims to understand the level of stigma of young and middle-aged MHD patients and the impact of social, cultural and hemodialysis indicators on patients’ stigma to formulate anti-stigma intervention measures and psychological health programs for young and middle-aged MHD patients.

## Materials and Methods

### Patients and Setting

This study was conducted as a cross-sectional descriptive study. A convenience sampling method was used to collect information from 97 young and middle-aged patients at Shanghai Sixth People’s Hospital between November 2020 and February 2021. All patients were given adequate pain assessment and analgesic treatment before dialysis and during catheter maintenance operations. The inclusion criteria were as follows: (1) patients who were 18∼59 years old and had regular dialysis for more than 3 months; (2) patients who had no cognitive impairment; and (3) patients who consented to voluntary participation in the study. The exclusion criteria were as follows: (1) patients with other serious diseases or tumors; (2) patients with mental illness preventing their cooperation; or (3) patients diagnosed with depression and receiving antidepressant treatment.

### Measurements

An 11-item questionnaire on the demographic characteristics of medical staff was administered. The questionnaire collected information on age, gender, education level, marital status, occupation, annual household income, family residence, disease-related data (such as primary diseases and comorbidities), and vascular access types.

The Social Impact Scale (SIS) is part of a universal scale for evaluating the stigma associated with two or more types of diseases; it was compiled by Fife and Wright (2020) and translated into Chinese (Taiwan) by Pan (Pan et al., 2007). The scale includes 24 items across four dimensions (dimension I, social rejection; dimension II, economic insecurity; dimension III, internalized shame; and dimension IV, social isolation). The scale adopts the following four-point scoring method: (4) strongly agree, (3) agree, (2) disagree, and (1) strongly disagree. The total score is the sum of scores for the four dimensions. The higher the score is, the more severe the stigma. In this study, the Cronbach’s α coefficients of each dimension of the scale were between 0.85 and 0.90. Regarding hemodialysis indicators, Kt/V is an important index to evaluate the effect of toxin clearance in dialysis patients. The higher the value is, the better the treatment effect. In our study, Kt/V was an important predictor of patients’ treatment status and physical status. Kt/v ≥ 1.2 is defined as adequate dialysis, and Kt/v < 1.2 is defined as inadequate dialysis (Breitsameter et al., 2012). In addition to (Kt/v), the hemoglobin, serum calcium, phosphorus and potassium levels of the subjects were also assessed as medical factors of MHD in our study. These indicators were statistically analyzed by the information management software system of Shanghai Sixth People’s Hospital.

### Ethical Considerations

This study was approved by the Hospital Ethics Committee of Shanghai Jiao Tong University Affiliated Sixth People’s Hospital [No. 2021-KY-064(K)]. Consent for participation in the research study was obtained from all patients in written form. The study was conducted in compliance with the Declaration of Helsinki.

#### Data Collection

The participants were recruited from the outpatient clinics of a hemodialysis center of a municipal hospital in Shanghai. Before the study, a research assistant explained the purpose of the study and obtained written informed consent from each participant. The participants were guided by researchers to fill out the electronic questionnaire or paper questionnaire according to their own situations. The data from the web-based questionnaires and paper questionnaires were anonymously submitted to the central database. Only the research team had access to the central database for academic research. The answers were checked immediately after questionnaire completion, and missing items were identified and completed to ensure the validity of the questionnaire. Finally, 105 questionnaires were distributed, and 97 valid questionnaires were collected for an effective recovery rate of 92.3%.

#### Statistical Analysis

After the elimination of invalid questionnaires, the data were entered and numbered. SPSS 26.0 was used for the statistical analyses. We used descriptive statistical analyses (measurement data with the mean and standard deviation). Count data were described as the frequency and composition ratio to describe the general data. Comparisons between the average SIS score and the norm were performed by independent samples *t* test. The effects of the general conditions on the stigma score of young and middle-aged MHD patients were compared by independent samples *t* test and single-factor analysis of variance (ANOVA). Multiple regressions were used to further analyze the predictive effect of the general data on the stigma of the population. All statistical tests were performed bilaterally, and *P* < 0.05 was considered statistically significant.

## Results

Table 1 shows the sociodemographic characteristics of the sample. The sample comprised 97 participants, including 73 males (75.3%) and 24 females (24.7%). The majority of participants (87.6%) lived in the city. The mean duration of hemodialysis was 4.19 ± 3.69 years.

**TABLE 1:** Participant characteristics.

*N* = 97	M ± SD or n (%)
Age (years)	47.55 ± 8.07
Female	24 (24.7)
Male	73 (75.3)
**Educational background** Primary school education	3 (3.1)
Junior middle school education	37 (69.8)
High school/college education	45 (46.4)
Undergraduate or above	21 (21.7)
**Occupation** Employed	34 (35.1)
Unemployed	63 (64.9)
**Family residence** Lived in a city	85 (87.6)
Lived in a township	12 (12.4)
**Annual household income** < $1800	7 (7.2)
1800–4000	11 (11.3)
4000-10000	43 (44.3)
≥ $10000	36 (37.2)
**Insurance status** Uninsured	5 (5.2)
Public insurance	92 (94.8)
Hemodialysis duration (years)	4.19 ± 3.69
**Primary disease** Chronic glomerulonephritis	24 (24.7)
Hypertensive nephropathy	21 (21.7)
Diabetic nephropathy	35 (36.1)
Other disease	17 (17.5)
Vascular access type, Central venous catheter	22 (22.7)
Autologous arteriovenous fistula	75 (77.3)
**Hemodialysis indicators** KT/V < 1.2	55 (56.7)
KT/V ≥ 1.2	34 (43.3)

The total SIS score was 43.69 ± 8.62 points, which was at the middle level. The four dimension scores were as follows: social rejection (13.20 ± 4.18), economic insecurity (6.29 ± 2.11), internalized shame (9.65 ± 2.97), and social isolation (14.58 ± 3.99). The average item score was 1.82 ± 0.36. The SIS scores of the young and middle-aged MHD patients were lower than the norm for China (Zheng et al., 2020), and the differences were statistically significant (*P* < 0.05). The details are shown in Table 2. The homogeneity of variance was tested by Levene’s test, and all groups of samples were consistent with the homogeneity of variance (*P* > 0.1). The results of the one-way ANOVA showed that the SIS scores of young and middle-aged MHD patients varied significantly by age, gender, family residence, annual household income, duration of hemodialysis and Kt/V value (*P* < 0.05), as shown in Table 3.

**TABLE 2:** Social impact scale scores of young and middle-aged maintenance hemodialysis patients.

Item	*N*	Score	Norm	*N*	*t*	*P*
Total SIS score	97	43.69 ± 8.62	56.80 ± 13.13	222	−9.01	0.000
Dimension I—social rejection	97	13.20 ± 4.18	19.71 ± 5.16	222	−10.95	0.000
Dimension II—economic insecurity	97	6.29 ± 2.11	7.70 ± 2.31	222	−5.41	0.000
Dimension III- internalized shame	97	9.65 ± 2.97	12.14 ± 3.17	222	−6.57	0.000
Dimension IV- social isolation	97	14.58 ± 3.99	17.25 ± 4.19	222	−5.31	0.000

**TABLE 3:** Comparison of SIS scores between young and middle-aged MHD patients by demographic characteristics.

Item	*N*	Score	Statistic	*P*
Age			*t* = 3.163	0.002
18∼44	34	47.32 ± 8.15		
45∼59	63	41.73 ± 8.39		
Gender			*t* = 2.303	0.023
Male	73	44.82 ± 8.32		
Female	24	40.25 ± 8.80		
Marital status			*F* = 0.909	0.406
Married	71	43.07 ± 8.41		
Unmarried	17	46.12 ± 8.00		
Divorced	8	44.89 ± 12.46		
Educational attainment			*F* = 1.150	0.333
Primary school	3	36.67 ± 8.33		
Junior high	37	45.43 ± 9.30		
Senior high school and technical secondary school	45	43.60 ± 9.26		
Bachelor’s degree and above	21	42.57 ± 6.07		
Working status			*t* = 2.05	0.042
On-the-job	34	46.42 ± 8.75		
Not working	63	42.69 ± 8.42		
Family residence			*t* = 0.100	< 0.001
City	85	42.55 ± 7.94		
Township	12	51.75 ± 9.31		
Annual household income			*F* = 2.747	0.047
< $1,800	7	50.80 ± 11.73		
1,800–4,000	11	46.77 ± 6.61		
4,000–10,000	43	43.95 ± 8.47		
> $10,000	36	41.35 ± 8.40		
Method of payment			*t* = 0.934	0.353
Medical insurance	92	43.50 ± 8.70		
Private expense	5	47.20 ± 6.87		
Complications			*t* = 0.219	0.827
No	85	43.95 ± 8.26		
Yes	8	43.25 ± 12.79		
Duration of hemodialysis			*t* = 2.622	0.041
≤ 2 years	33	43.12 ± 6.60		
2∼4 years	24	42.25 ± 9.09		
4∼6 years	11	50.10 ± 13.14		
6∼8 years	8	43.25 ± 6.71		
> 8 years	12	39.24 ± 8.66		
Original disease			*F* = 1.190	0.318
Chronic glomerulonephritis	24	44.00 ± 9.56		
Hypertensive nephropathy	21	41.48 ± 9.70		
Diabetic nephropathy	35	43.34 ± 7.48		
Miscellaneous	17	46.71 ± 7.87		
KT/V(mmol/L)			*t* = 2.545	0.013
< 1.2	55	45.02 ± 8.92		
≧1.2	34	40.38 ± 7.33		
HGB(mmol/L)			*t* = −1.231	0.222
Not anemic	42	44.57 ± 9.99		
Anemic	41	42.20 ± 7.37		
CA(mmol/L)			*t* = 0.109	0.913
< 2.25	25	43.56 ± 8.60		
2.25∼2.75	58	43.33 ± 8.99		
P(mmol/L)			*t* = 5.578	0.565
0.97∼1.61	68	43.66 ± 9.43		
> 1.61	15	42.20 ± 5.32		

The SIS scores of young and middle-aged MHD patients were used as the dependent variable, and the variables with significant differences in the univariate analysis as well as occupational status were used as independent variables to perform multiple linear regression analysis. Continuous variables were input according to the original value (age), and classified variables were treated as dummy variables. The results showed that when age, family income, gender, working status and the duration of hemodialysis were entered into the regression model (*P* < 0.05), they explained 39.6% of the variation in stigma (R^2^ = 0.464, adjusted R^2^ = 0.396). As shown in Table 4, younger MHD patients had more stigma than older MHD patients, and a statistically significant difference was detected (*P* < 0.05). MHD patients with lower income had a significantly higher level of stigma (*P* < 0.05). In addition, male young and middle-aged MHD patients who were working had a higher stigma score. The duration of hemodialysis positively predicted stigma in young and middle-aged MHD patients.

**TABLE 4:** Multivariate linear regression analysis of the influencing factors for stigma in young and middle-aged MHD patients.

Variable	B	SE	Beta	*t*	*P*
Age	−2.176	0.495	−0.401	−4.398	< 0.001
**Working status**					
On-the-job	5.273	1.869	0.273	2.821	0.006
**Gender**					
Male	5.097	1.776	0.253	2.870	0.005
**Annual household income**					
$1,800–4,000	−2.303	3.909	−0.092	−0.589	0.557
$4,000–10,000	−4.114	3.448	−0.238	−1.193	0.237
>$10000	−9.428	3.539	−0.532	−2.664	0.009
**Duration of hemodialysis (years)**					
2∼4	1.219	1.851	0.063	0.658	0.512
4∼6	7.184	2.532	0.276	2.837	0.006
6∼8	3.747	2.801	0.125	1.338	0.185
>8	0.956	2.466	0.038	0.387	0.699

*R^2^ = 0.464, adjusted R^2^ = 0.396, F = 6.765, P < 0.05.*

## Discussion

### Stigma Among Young and Middle-Aged MHD Patients Is at a Medium Level

This study aimed to determine the stigma status of young and middle-aged MHD patients and identify its influencing factors in China. The results of this study showed that the total SIS score of young and middle-aged MHD patients was 43.69 ± 8.62, which was at the middle level. The probable causes of the middle level of the stigma found in this study are as follows. First, MHD patients often experience negative physical changes, such as skin itching, edema, nausea, vomiting and the use of indwelling venous catheters, which affect their daily communication. These effects can lead to negative emotions in patients’ work and lives. Second, in China, hemodialysis treatment can be completed only in the hospital, and patients must come to the hospital 2–3 times a week, making it difficult for them to go out for long periods of time; this affects their work and study, causes them to worry about their future and even makes them feel discriminated against and prejudged (Novick et al., 2018). Traditional Chinese values highlight moral excellence and self-cultivation of harmony within the self, family and society (Sun et al., 2018). Common stereotypes of the danger and unpredictability of hemodialysis treatment directly challenge these cultural norms, leading to stigma. Finally, patients who receive hemodialysis have complex medical regimens and challenging dietary restrictions. Some dialysis patients experience body image disorders and a decline in sexual function, which aggravates the experience of stigma (Álvarez-Villarreal et al., 2019). Therefore, the vast majority of patients experience varying degrees of stigma, which is consistent with other research results in China. In this study, the SIS score of the respondents was lower than the norm for China, and the difference was statistically significant (Zheng et al., 2020). The reason may be related to regional cultural and economic differences. MHD patients require long-term dialysis treatment, and the disease cannot be cured, which easily causes patients to feel helpless and discriminated against from an economic perspective (F Yang et al., 2015). In our study, the patients mainly came from Shanghai, which is an economically developed city in China. The medical conditions and treatment service level of patients in this city are significantly better than those in other parts of China. Most patients in this study had medical insurance, which reduced the economic burden on patients.

Among the four dimensions of stigma, the average item scores from high to low were for the dimensions of economic insecurity, social isolation, internalized shame and social rejection, reflecting that the main source of stigma in young and middle-aged MHD patients is economic problems, followed by social isolation. The reasons may be as follows: young and middle-aged MHD patients transition from being the backbone of society to being heavy economic burdens on individuals or families. Medical costs such as hemodialysis and the negative impact on employment caused by persistent diseases may increase the economic pressure of patients and cause greater stigma. A previous study showed that the self-perceived burden of hemodialysis patients was heavy and that 65.6% of patients reported moderate to severe levels of burden (Ma et al., 2020). In addition to the direct impact of disease, the role of patients’ inner experience, such as social isolation, in the process of stigma addition cannot be ignored. In this study, 36% of patients agreed with the item “I distinguish myself from healthy people,” 43% of patients agreed with the item “Because of my disease, sometimes I feel useless,” and 25% of patients endorsed the item “I feel lonelier than ever.” MHD patients need dialysis treatment 2–3 times a week and cannot go out for long periods of time. This makes patients worry about their future careers and social lives, and they feel left out of social activity due to losing the opportunity to go out with his family and friends. In addition, some studies have shown that after dialysis, patients often have symptoms such as drowsiness and decreased desire to work, which seriously affect their social lives. The combination of the above factors promotes patients’ sense of social isolation, which is accompanied by inferiority and depression (Ma and Li, 2016). According to American scholars, approximately 44% of MHD patients diagnosed with depression receive antidepressant treatment, and 3% receive psychiatric consultation (Pena-Polanco et al., 2017). The above loneliness and social isolation are also important reasons for psychological problems and poor quality of life in young and middle-aged MHD patients. Therefore, while paying attention to patients’ inner experiences and helping them develop a healthy mentality, we should also emphasize the popularization of public knowledge to improve the acceptance of MHD patients and to reduce the social isolation of young and middle-aged MHD patients.

### Factors Influencing Stigma in Young and Middle-Aged MHD Patients

Age: The results of this study showed that the stigma scores of young MHD patients (18–44 years old) were significantly higher than those of the middle-aged group (*P* > 0.05), indicating that the stigma among young patients was stronger than that among older patients, which was basically consistent with the results of other studies (Millum et al., 2019; Link and Phelan, 2001). Most young MHD patients have unstable psychological and economic statuses, for example, because they are in school or job hunting; therefore, they often lack life experience and have poor acceptance of sudden diseases. Gredig and Bartelsen-Raemy (2017) also found that in Switzerland, the younger the patients with diabetes were, the more unfair they perceived the treatment to be, and the more exclusion they experienced. Social stereotypes are also common. In social interactions, young MHD patients often form a sharp contrast with most of their peers in terms of health status, which leads to a very large psychological gap and pressure. Therefore, compared with middle-aged patients, young patients are more likely to have a sense of inferiority, to feel useless and to feel abandoned.

Gender: This study found that male patients with MHD had higher stigma scores than women, which was basically consistent with the results of another study (Chatmon, 2020). The reasons may be as follows. Most men are the backbone of their families, playing a supportive role in the family and undertaking important family and social expectations and social roles. A decline in self-realization can be caused by disease. The loss of employment opportunities leads to deterioration in one’s economic conditions, a decline in family and social status, and a lack of dignity. A previous study also showed that female patients’ high acceptance of disability could be related to their more frequent use of talk to dispel their negative emotions, seek solutions and thus gain more social support than men (Lee et al., 2021).

The duration of hemodialysis: In this study, the duration of hemodialysis was another influencing factor of stigma in young and middle-aged MHD patients. The stigma scores of hemodialysis patients with a short duration of hemodialysis were lower than those of patients with a long duration, which is consistent with the results of Kryseana’s survey of hospitalized diabetic patients (Harper et al., 2018). As hospital admission is a negative, stressful event, multiple admissions for hemodialysis treatment can increase the psychological burden on patients, enhance the sensitivity of patients to external stimuli, and thus make them more prone to stigma. Rather than being in the early stage of another disease, hemodialysis patients are in the end stage of kidney disease and therefore need to gradually accept the disease, which can easily increase stigma, consistent with the conclusion of this study. The reason may be that with the progression of the disease, the complications of hemodialysis in patients are gradually revealed, and renal function is further reduced. Hemodialysis is only a means to delay the progression of the disease, but it cannot prevent the deterioration of health. With time, patients’ physical symptoms are gradually aggravated, and the sense of shame increases. Different from young and middle-aged MHD patients with hemodialysis durations of 4–6 years, those with hemodialysis durations of more than 6 years had a lower sense of stigma. In the sample investigated in this study, those with a hemodialysis duration of more than 6 years were mostly middle-aged patients, and there were more males than females; therefore, they had a lower sense of stigma.

Income: The results of this study showed that the stigma level of high-income people was lower than that of low-income people. The reason may be that people with higher incomes are able to meet the requirements to maintain their own lives, social networking and image. Therefore, the economic anxiety and stress caused by medical expenses are more manageable. Meanwhile, people with lower incomes are less resilient to medical expenses. Research by Smith et al. (2020) also showed that long-term medical costs can increase the economic burden on patients’ families and make patients feel guilty and more sensitive to stigma. On the other hand, patients with a higher income have a higher degree of self-worth in society, which is relatively easily recognizable by the outside world. These patients experience fewer discrimination events and thus have fewer internal stigma experiences (Schabert et al., 2013).

Work: The results of this study showed that the stigma level of working young and middle-aged MHD patients was higher than that of non-working patients. The reason may be related to the fact that employed patients not only face task pressure from the work itself but also must deal with the extra social pressure caused by their illness. The social relations of employed patients are often more complex because they need to deal with interpersonal events with leaders, colleagues and customers. In this case, they face greater risks of exposure to diseases or discrimination. In addition, in this study, the average age of the employed patients was lower than that of the unemployed patients, and the psychological characteristics and burden of the younger patients also had a partial impact on stigma. Therefore, the results showed that the stigma level of employed patients was higher than that of unemployed patients.

Kt/V: The stigma score in patients with adequate hemodialysis was lower than that in patients with inadequate hemodialysis in this study, and the results of single-factor ANOVA showed that the difference was statistically significant (*P* < 0.05). A previous study found that dialysis adequacy was positively correlated with commonly used nutritional indicators (Kalantar-Zadeh et al., 2003) and that those with insufficient dialysis were more likely to suffer from malnutrition, which is an important factor in the decline in quality of life and the increase in mortality and hospitalization rate among MHD patients. Therefore, inadequate hemodialysis aggravates the social isolation of MHD patients and increases the stigma experienced by patients. However, hemodialysis adequacy could not be entered into the regression equation, which may be related to the sample size of our study. Therefore, whether dialysis adequacy is an influencing factor of stigma in young and middle-aged MHD patients needs further research.

### Limitations and Prospects

The study has a few limitations. First, this study was a preliminary study using a convenience sample of MHD patients from a hospital in Shanghai, covering a limited number of patients. Second, the influencing factors included in this study were not comprehensive enough, and more social psychological factors still need to be explored. In the future, multicenter studies over longer observation times and even on older patients need to be carried out.

## Conclusion

This study suggested that age influences the stigma of MHD patients and that younger MHD patients require much more care and consideration regarding their stigma. Furthermore, the results confirmed that income influences the stigma of young and middle-aged MHD patients. Young and middle-aged MHD patients with lower income should be considered worthwhile targets for preventing stigma and ensuring psychological health. Additionally, MHD patients with more hemodialysis experience are much more stigmatized than those with less hemodialysis experience. In particular, anti-stigma programs should be directed at experienced patients with a longer duration of hemodialysis.

## Data Availability Statement

The original contributions presented in the study are included in the article/supplementary material, further inquiries can be directed to the corresponding author/s.

## Ethics Statement

The studies involving human participants were reviewed and approved by the Hospital Ethics Committee of Shanghai Jiao Tong University Affiliated Sixth People’s Hospital [No.2021-KY-064(K)]. The patients/participants provided their written informed consent to participate in this study.

## Author Contributions

All authors made significant contributions to the work reported, whether in the conception, study design, execution, acquisition of data, analysis and interpretation, or in all these areas, participated in drafting, revising or critically reviewing the article; gave final approval of the version to be published; agreed on the journal to which the article has been submitted; and agreed to be accountable for all aspects of the work.

## Conflict of Interest

The authors declare that the research was conducted in the absence of any commercial or financial relationships that could be construed as a potential conflict of interest.

## Publisher’s Note

All claims expressed in this article are solely those of the authors and do not necessarily represent those of their affiliated organizations, or those of the publisher, the editors and the reviewers. Any product that may be evaluated in this article, or claim that may be made by its manufacturer, is not guaranteed or endorsed by the publisher.
